# Mutations that enhance evolvability may open doors to faster adaptation

**DOI:** 10.1038/s41467-023-41914-2

**Published:** 2023-10-09

**Authors:** C. Brandon Ogbunugafor

**Affiliations:** 1 Department of Ecology and Evolutionary Biology, Yale University, New Haven, CT USA; 2 Santa Fe Institute, Santa Fe, NM USA

**Keywords:** Molecular evolution, Evolutionary theory

## Abstract

A recent study demonstrated the existence of mutations that facilitate access to efficient evolutionary solutions. Here I discuss the implications of this finding and the potential to open a new chapter in the study of evolvability.

The concept of evolvability has a relatively recent and complex history. As a general idea, it appears in the scientific literature in the 1990s, peaking and plateauing in mentions in the early 2010s (Fig. 1)^1^. It is based on the question of whether the capacity to evolve can itself evolve. One of its chief challenges to broader adoption involves its many interpretations, which can confound how it is measured and operationalized^2^. One definition refers to the general ability of replicators to respond to the force of selection^3^. Another slightly more detailed definition suggests that evolvability involves the capacity of populations to generate adaptive variation that promotes evolution by natural selection^4^. Using these and many other framings, evolvability can be the product of the same information as any trait and is then subject to forces of evolution (e.g., mutation, migration, selection, drift).

**Fig. 1:**
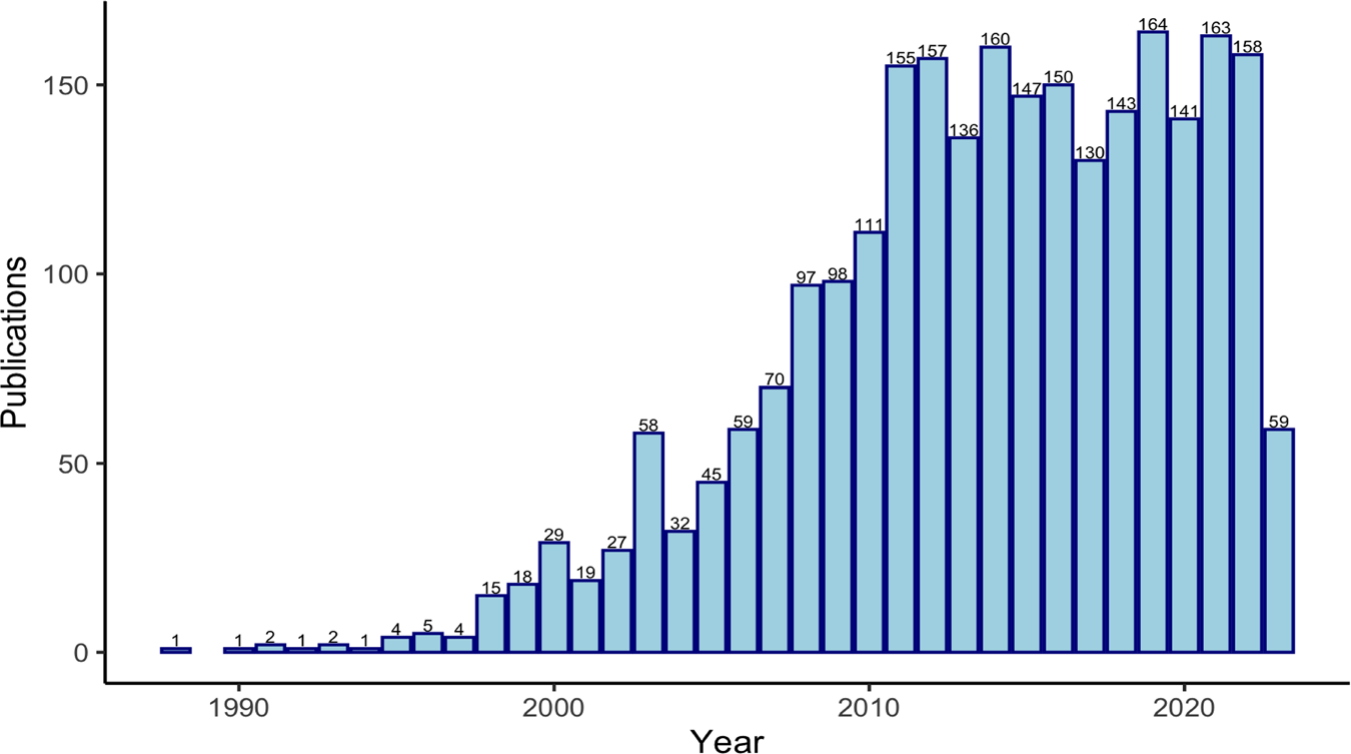
“Evolvability” in the literature through time. The Web of Science database was searched on June 2, 2023, using the search term “evolvability.” The initial search returned 3172 results. Results were then limited to articles, review articles, book chapters, book reviews, and books, reducing the number to 2562. The results were then downloaded using the “Analyze Results” feature of the Web of Science, which summarized the number of studies published yearly since 1988.

## Evolvability and its modern controversies

When considered this way, a library of questions surface: is evolvability a complex trait that varies (in magnitude or character) across and within taxa? Which sorts of ecologies select for or against evolvability? What is the molecular machinery that underlies it? When we consider the possible answers, the intrigue with the phenomenon becomes clear: evolvability can reframe many aspects of interpreting, measuring, and predicting evolution. This explains why it has been present in discussions surrounding the extended evolutionary synthesis, an attempt to integrate newer theoretical ideas (e.g., plasticity,, epigenetics, and others) into the central canon^5^. Further, it has implications for any field where understanding the capacity to evolve might be relevant, from biomedicine to the study of technological and cultural evolution.

While the concept remains popular among evolutionary theorists, we can fairly ask about its broader relevance. Arguably, evolvability has been trapped in a corner of evolutionary theory, where its potential to improve our understanding is greater than its impact on how evolutionary biologists approach critical questions. Relatedly, what practical problems does its appreciation help us solve or understand? To many in the field, the answers to these questions are frustratingly inadequate and have limited evolvability’s inclusion in the center of canon in evolutionary biology, as was the hope of many who championed its implementation into the extended evolutionary synthesis.

**Fig. 2:**
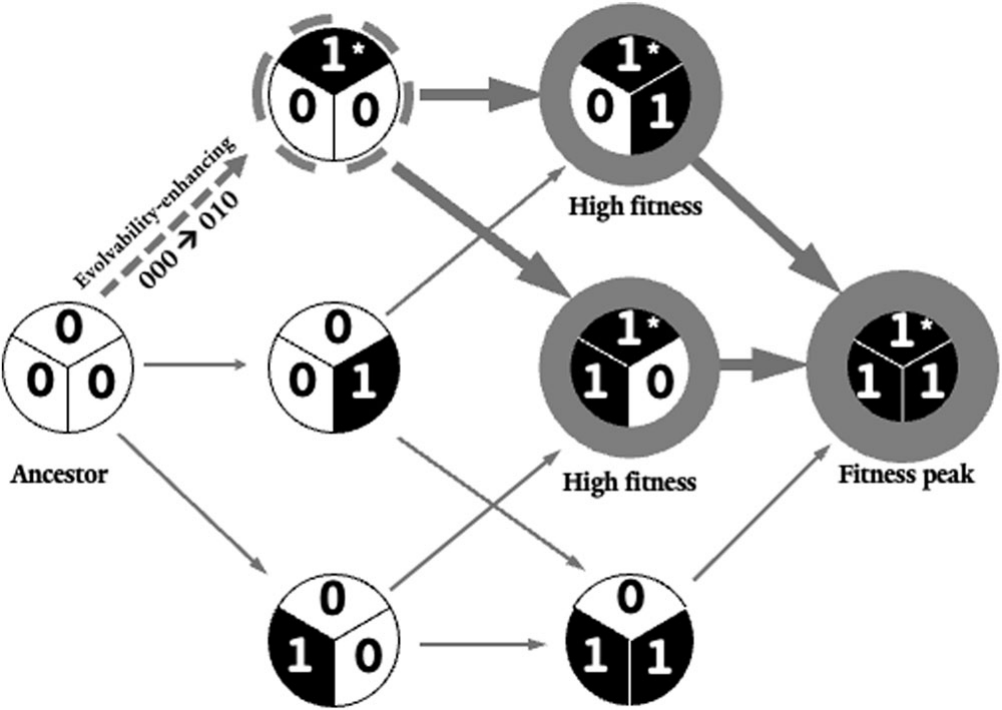
A simplified conceptual depiction of an example of evolvability-enhancing mutations. In this scenario, with binary representation, [0] and [1] corresponding to the presence and absence of a mutation. In a standard hypergraph description of a combinatorially-complete set of mutations, an evolvability-enhancing mutation is present in high-fitness genotypes (large grey circles). The mutation at the second locus (1*) is an evolvability-enhancing mutation because all subsequent mutations on a genetic background that contain it are relatively high fitness alleles, including the fitness peak (111). Weighted arrows correspond to accessible trajectories to the peak. Note that evolvability enhancing mutations need not increase fitness on their own, but rather, provide access to high fitness sections a fitness landscape.

In a new study published in *Nature Communications*, Andreas Wagner utilizes large data sets and computational tools to offer provocative ideas about the frequency, phenotypic effects, and evolutionary consequences of mutations that facilitate access to beneficial mutations and more efficient searches for fitness peaks^6^. Specifically, Wagner identifies “evolvability-enhancing” mutations that create a genetic background where subsequent mutations are more likely to be beneficial relative to mutations acquired on an ancestral background by virtue of their average mutational neighbor being of higher fitness (Fig. 2). In addition, such backgrounds facilitate the search for novel adaptations.

## The search for mutations that promote evolvability

Wagner analyzed data from two different large data sets: All 20 amino acid variants at each of three sites (roughly 8000 variants) in a protein in the *Escherichia coli* toxin-antitoxin system, and 4000 variants of a transfer RNA (tRNA) from *Saccharomyces cerevisiae* across 10 nucleotides. Using two completely different biomolecules is critical because Wagner aimed to identify a true signature associated with increased evolvability rather than one peculiar to a certain information space.

In this way, Wagner was able to address the question of whether biological landscapes contain more evolvability-enhancing mutations than would be expected by chance. He found that both the protein and RNA empirical landscapes had more than their randomized counterpart, suggesting a pattern that is unique to biological systems.

Next, Wagner examined whether adaptive trajectories containing evolvability-enhancing mutations lead to higher fitness sections of fitness landscapes. Using stochastic simulations, Wagner found that adaptive walks that contained evolvability-enhancing mutations were associated with significantly higher fitness gain in both the protein and RNA fitness landscapes relative to those without evolvability-enhancing mutations.

What are the implications? One is that these evolvability-enhancing mutations offer a means through which we can consider how evolvability is constructed bit by bit from the mutations that compose fitness landscapes. Another implication is that evolvability isn’t only observed at the organismal or population scales but can be measured at the level of individual traits, genes, or mutations.

Furthermore, the study emphasizes the importance of both big data and classical conceptual instruments like the fitness landscape in providing mechanistic nuance to abstract concepts like evolvability. The use of fitness landscapes—an analogy for genotype-phenotype space where the forces of evolution move genotypes up “fitness peaks” and down “fitness valleys”^7^—as a model for studying evolvability is not new. A seminal study by Ancel and Fontana^8^ explored RNA sequences to propose principles for how characteristics of high-dimensional spaces were models for basic questions in evolvability. In the last two decades, many others have followed suit. Moreover, while these studies were foundational, helping to build the modern field of evolvability as we know it, many aspects remained underexamined. For example, while studies have demonstrated evidence for how evolvability relates to biophysical features of proteins^9^, fewer have attempted to formalize differences in evolvability in terms of reproducible computational rules, analytical expressions, or metrics that quantify the evolvability of replicators. Wagner’s latest work attempts to modernize the study of evolvability by highlighting how it can be wired into biological information spaces of various kinds.

The author acknowledges that while biological fitness landscapes contain more evolvability-enhancing mutations than in silico randomized landscapes, that does not mean that their presence (in proteins and RNA) is driven by adaptive evolution. Said differently, the “enhancing” part of “evolvability-enhancing” should not be interpreted in a teleological or adaptationist sense: there is no evidence that they exist in order to enhance evolvability. Their existence might be an artifact of features of genotype-phenotype space, where epistasis (the nonlinear interaction between the effects of mutations) influences the shape and topography of fitness landscape and adaptive trajectories^7,10^.

In one sense, whether these evolvability-enhancing have an adaptive origin or not isn’t so significant: even if they are artifacts, we should study them if they play a role in molecular evolution. On the other hand, if they are mere byproducts of some feature of how biomolecules are constructed, then some of the intrigue is diminished—they may be less relevant for the bigger question of how evolvability evolves.

More generally, one can reasonably ask whether the existence of evolvability-enhancing addresses any large gap or conflict in evolutionary biology? It is not so clear. Further, the invocation of a new term and description for a type of mutation in fitness landscapes should (eventually) be formalized in (and reconciled with) the grammars of theoretical population genetics, which contains many decades of work on mutation effects in populations^11^.

## Applications to public health and bioengineering

These issues aside, Wagner’s findings offer an important new lens on how genotype-phenotype space is constructed, and by extension, how evolution happens at the molecular level. The observed differences in the frequency and effect of evolvability-enhancing mutations in RNA and protein implore us to search for informational and biophysical explanations: are there features of spaces that facilitate more evolvability-enhancing mutations? Even more, one can ask whether evolvability-enhancing mutations can be engineered into replicators–biological, artificial, or cultural—towards controlling the pace and direction of evolution.

The most proximal application of evolvability-enhancing mutations might reside in the public health realm. For example, surveillance for troublesome mutations should not only include “escape” variants (for vaccines) or other variants of concern^12^, but also those mutations that facilitate the evolution of other more troublesome variants (as suggested by evolvability-enhancing mutations). These notions have resonance with recent developments in *Vibrio cholerae*, where certain mutations provide the genotypic context for virulence genes to express their deadly phenotypic effects^13^. And even closer to evolvability-enhancing mutations are the existence of “epistatic ratchets^14^” and “pivot mutations” (the latter discussed in malaria)^15^. Both are examples of mutations that interact with other mutations (via epistasis) and constrain evolution. In light of this, the existence of evolvability-enhancing mutations can contribute to the growing movement to describe the effects of disease-associated mutations with respect their performance across varied contexts, what phenotypic (clinical) outcomes they can foster, and the evolutionary consequences they facilitate. Further, this knowledge can be applied to bioengineering efforts to build biological systems to be more or less evolvable.

We should be encouraged by attempts to add more mechanistic detail to the study of evolvability, starting with the individual mutations that may give it a boost. This study highlights how large data and new technologies, in the context of theoretical insight, may walk us toward a more rigorous look at the many engines that drive how adaptive evolution happens in the manner that it does.

## Data Availability

The data corresponding to the literature search in Fig. 1 can be found the Web of Science.We have made the data available at Github: https://github.com/OgPlexus/evolvabilitynews1.
